# Overview of advanced non-small-cell lung cancer treatment in Mexico

**DOI:** 10.1186/1753-6561-2-s2-s2

**Published:** 2008-09-24

**Authors:** Víctor Lira Puerto

**Affiliations:** 1 Departamento de Oncología Médica, Hospital ABC México, Sur 136 No 116 Col. Las Américas, 01120 México DF, México and Centro Medico Siglo XXI, Av. Cuauhtmoc 330 Col. Doctores 06720 México DF, México

## Abstract

**Background:**

Lung cancer is the leading cause of cancer-related deaths among males and the second among females. The importance of lung cancer is a major public health problem and there is a need to find effective therapies for its management. Erlotinib has been approved to treat non-small-cell lung cancer. The author's experience in the use of erlonitib in lung cancer patients in Mexico City is described below.

**Methods:**

The series includes 17 consecutive patients treated for advanced lung cancer. All patients had measurable disease. Treatment continues until disease progression or significant toxicity occurs. Among patients, adenocarcinoma was the most common tumor histology, followed by bronchioloalveolar tumor, and epidermoid carcinoma. Nine patients received erlotinib as first-line therapy. Of the remaining 8 patients, 4 had undergone surgery, 2 had received chemotherapy, and 2 had received combined chemotherapy and radiotherapy.

**Results:**

Four patients achieved complete remission of the disease, and 7 showed partial response. Five subjects experienced disease progression, and one patient showed stable disease. The most significant cases were two non-smokers women with bronchioloalveolar cancer, who remain in complete remission after erlotinib treatment. A non-smoker male patient with adenocarcinoma histology, who rejected chemotherapy and radiotherapy, it remains in complete remission after 15 months of treatment. A man with epidermoid carcinoma, with previous surgery and treated with chemotherapy and radiotherapy, with tumor recurrence, showed a complete 15-month remission with erlotinib. It was observed clinical response due to treatment with erlotinib despite the tumor histopathology, but therapeutic response was better in patients without smoking history. The most common adverse events associated with erlotinib therapy were dermatologic. After discontinuing treatment for a short period, patients were again given erlotinib without experiencing toxic effects. Hepatotoxic side effects associated to erlotinib were mild and reversible.

**Conclusion:**

Data from this small series of patients support findings reported in the literature. Female non-smokers showed the best therapeutic response to erlotinib treatment. Erlotinib could be considered as a first-line therapeutic option in elderly patients with locally advanced or metastatic lung cancer, or in women with adenocarcinoma.

## Background

Lung cancer is the most common cause of death from cancer in both men and women worldwide [1]. Among males it is the leading cause of cancer-related deaths, while among females it is second only to breast cancer [2]. In Mexico, crude mortality due to lung cancer in 1979 was 5.01 per 10^5 ^people [3]. Data from a recently published study place emphasis on lung cancer burden in Mexico [4]. From 1998 to 2004 approximately 400,000 deaths from cancer were reported in that country, with lung cancer accounting for 11.5% of them. Mean mortality rate from this neoplasm was 8.9 per 10^5 ^people among males and 4.1 per 10^5 ^people among females. The highest overall mortality rates from lung cancer were noted in the northern regions of the country, where the most industrialized states are located. However, if women are considered individually, the highest rates were found in the central states. Days of life potentially lost due to lung cancer were nearly 260,000 among men and approximately 133,000 among women. These figures underscore the importance of lung cancer as a major public health problem and the need to find effective therapies for its management.

Erlotinib is a new potent inhibitor of the epidermal growth factor receptor (HER1/EGFR) which has been approved to treat non-small-cell lung cancer (NSCLC). The author's experience in the use of erlonitib in lung cancer patients in Mexico City is described below.

## Methods

### Population

The series includes 17 consecutive patients treated for advanced lung cancer from November 2005 to November 2007. Patients' median age is 64 years (range 47–82). All patients had measurable disease, and 11 of them had no smoking history. Nine patients were female. The first 10 patients of the series were enrolled according to the protocol for the Expanded Access Program of TRUST (TaRceva lUng cancer Survival Treatment), developed in 52 countries after results of the phase III trial referred to as BR.21 were available [5,6]. The following 7 patients were included in this series out of protocol and were selected by sex and tumor histology. Median follow-up for all patients enrolled in the series was 9 months.

The TRUST study is a phase IV multicenter open-label non-randomized trial in patients with advanced nonresectable stage IIIB/IV NSCLC after failure of prior standard chemotherapy or radiotherapy regimen, or who are not candidates for cytotoxic chemotherapy, or refuse such therapy [6]. Performance status (PS) of enrolled patients could range from 0 to 3 as measured by ECOG score, and liver, renal and hematological function had to be adequate prior onset of treatment. The protocol is currently active although enrollment of patients is closed. Treatment continues until disease progression or significant toxicity occurs. Dose may be reduced in 50 mg decrements in the event of severe toxic effects. Until March 2007 data from 5908 patients were available, according to an intention-to-treat analysis.

### Histopathologic and clinical features: previous therapy

In this series patients were not randomly assigned; there was some degree of selection. Adenocarcinoma was the most common tumor histology (8 patients; 5 women, 3 men), followed by bronchioloalveolar tumor (6 patients; 4 women, 2 men), and epidermoid carcinoma (3 male patients). The greater number of women included in the series reflects selection by histopathological type of tumor. Clinical stage was III in 14 patients and IV in 3 patients.

Nine patients received erlotinib as first-line therapy (the 7 out-of-protocol patients and 2 patients enrolled according to protocol who refused standard chemotherapy as treatment). Of the remaining 8 patients, 4 had undergone surgery, 2 had received chemotherapy, and 2 had received combined chemotherapy and radiotherapy.

## Results

Four patients (23.5%) achieved complete remission of the disease, and 7 (41%) showed partial response. Five subjects experienced disease progression (29.4%), and one patient showed stable disease. Overall clinical benefit was 70.5%, and median duration of partial response was 6 months. It should be noted that these patients had some degree of selection and that response might not be so favorable in a non-selected population [7]. However, preliminary analysis of results from the TRUST trial shows that disease control rate (complete or partial response, or stable disease for at least 4 weeks) was 73% with erlotinib as first-line, 67% as second-line, and 66% as third-line therapy [6]. Therefore, the results in this series are similar to those of the overall population included in the protocol. These data could indicate that in patients selected according to tumor histology, smoking history and gender, among other factors, administration of erlotinib would provide the highest benefit, as suggested by some authors [7].

Among the most significant cases included in this series, two merit a particular discussion: 2 women aged 52 and 73 years, non-smokers, with bronchioloalveolar cancer, who remain in complete remission after 7 and 11 months of treatment, respectively. It should be noted that remission was observed during the first month of treatment with erlotinib. Monthly clinical as well as radiological and laboratory check-ups of enrolled patients allowed this finding [5,6]. Out-of-protocol patients in this series underwent similar check-ups as those required for the TRUST trial. In the two patients mentioned above, evidence of tumor activity was no longer observed within one month after treatment onset.

One of the cases included in this experience was an 80 year-old non-smoker male patient, with adenocarcinoma histology, who rejected chemotherapy and radiotherapy, and who was not a candidate for surgery due to advanced stage disease, and was therefore included in the TRUST protocol. The patient remains in complete remission after 15 months of treatment.

Another patient, a 73 year old man with epidermoid carcinoma, with previous surgery and treated with chemotherapy and radiotherapy, with tumor recurrence in the chest and neck on the opposite side of the primary tumor, showed a complete 15-month remission with erlotinib.

It should be noted that in this experience 5 patients were > 70 years old, and 3 of them showed complete remission.

### Therapeutic response according to tumor histopathology and smoking history

The evaluation of the relationship between tumor histopathology and therapeutic response to erlotinib in this series of patients, shows that 4 of the 8 patients with adenocarcinoma had positive therapeutic response. Five of the 6 patients with bronchioloalveolar carcinoma showed therapeutic response, and 1, stable disease. Clinical response was also observed in 2 of the 3 patients with epidermoid carcinoma.

As regards smoking status, therapeutic response was better in patients without smoking history (8 of the 11 patients responded and 1 remained with stable disease, compared to 3 responders among 6 smokers).

Despite the limited number of patients, these results are consistent with data from published literature [7].

### Adverse effects

A crucial issue in this series was determining the type and frequency of adverse effects associated with erlotinib therapy. The most common adverse events were dermatologic. Grade 4 rash was found in 2 patients (12%), grade 3 in 9 patients (53%), and grade 2 in 6 patients (35%). Data from TRUST protocol available to date show that skin rash is a highly common adverse effect, affecting 70% of patients. However, in most of them rash was grade 1 and 2 (58%) [6].

In 10 of the 17 patients included in the series, dose was reduced to 100 mg due to dermatologic side effects. In these cases, dose reduction did not appear to significantly affect therapeutic response. Data from the TRUST trial show that only 13% of patients required reduction of the dose due to adverse effects (mainly rash and/or diarrhea), and 6% experienced an erlotinib-related adverse effect leading to treatment withdrawal [6].

When looking at hepatic adverse effects, laboratory data showed grade 3 of toxicity in 1 patient, and grade 2 of toxicity in 2 patients. Toxic effects were detected through abnormal liver function tests in all cases, and no clinical effects were observed. After discontinuing treatment for a short period, liver function markers returned to normal. Patients were again given erlotinib at the initial dose without experiencing such toxic effects again. These data are consistent with those reported in previous studies showing that hepatotoxic effects are uncommon, and usually mild and reversible [8,9].

Fatigue occurred in 47% of patients. It is difficult to determine whether this symptom is related to therapy, since it may be another manifestation of the underlying disease.

The other frequent treatment-related adverse effect of erlotinib was diarrhea, which in some studies has occurred in up to 72% of patients [8]. In this series of patients, however, this condition was not so frequent, since only 2 patients developed grade 2 diarrhea (11%), which resolved with symptomatic treatment. This figure is slightly higher than the one found in the TRUST trial, where only 1% of enrolled patients experienced diarrhea [6]. A possible explanation for the data from this series of patients could be a reduction in the dose of erlotinib, already recommended. Still, patients did not develop diarrhea either when given the 150 mg dose, rather they showed dermatologic effects.

## Conclusion

In this experience with 17 patients, 5 of them were older than 70 years of age and 3 achieved complete remission.

Data from this small series of patients support findings reported in the literature regarding adenocarcinoma histology, which responds better to erlotinib than other histologic subtypes of NSCLC. Moreover, female non-smokers showed the best therapeutic response. Nevertheless, male patients with epidermoid histology may also be benefited.

Despite erlotinib dose reduction, disease-free period was considered to be relatively long.

In addition to its already established indications as second- and third-line therapy, erlotinib could be considered as a first-line therapeutic option in elderly patients with locally advanced or metastatic lung cancer, or in women with adenocarcinoma.

## Competing interests

Dr. Victor Lira Puerto has served on an advisory panel for Glaxo Smith Kline, Eli Lilly and Pfizer and has received grants/research support from Sanofi-Aventis and Roche.
